# Health economic evaluation of trans-tibial prosthetic suspension systems: a protocol for a pilot using an observational study and synthetic cohort

**DOI:** 10.1186/s12962-025-00611-1

**Published:** 2025-04-12

**Authors:** Leigh Clarke, Alan Shiell, Michael P. Dillon

**Affiliations:** 1 Discipline of Prosthetics and Orthotics, Department of Physiotherapy, Podiatry, Prosthetics and Orthotics, School of Allied Health, Human Services and Sport, La Trobe University, Melbourne, VIC 3086 Australia; 2 Department of Public Health, School of Psychology and Public Health, La Trobe University, Melbourne, VIC 3086 Australia

**Keywords:** Health economic evaluation, Cost-effectiveness, Cost-utility, Benefits, Time horizon, Prosthesis, Amputation

## Abstract

**Background:**

Health Economic Evaluations (HEEs) provide the necessary evidence of cost–benefit to inform policy and investment decisions. No HEEs have quantified the cost–benefit of passive suction (PS) vs vacuum assisted suction (VAS) suspension for trans-tibial prosthesis users. There are methodological challenges to conducting HEE in prosthetics given the benefit measures are not focused on the things most important to prosthesis users and funders, and the required time horizons are lengthy. To address these challenges, we propose a pilot study using two PROMIS instruments to measure benefits and trial the use of a Synthetic Cohort Method, to quantify the cost-effectiveness and cost-utility of PS and VAS suspension for people living with trans-tibial amputation.

**Methods:**

An observational study will measure the costs and benefits of PS and VAS suspension for trans-tibial prosthesis users using a Synthetic Cohort Method, a technique used in epidemiological modelling of life-time risks. Each intervention will include 3 sub-groups, representing prosthesis users in the first, second, or third year of the intervention since fitting. A prosthetic payor perspective will be taken, with data collected over a 1-year period and synthesised to reflect the costs and benefits over a 3-year time horizon. Benefits will be measured using two PROMIS instruments reported to best measure the benefits most important to prosthesis users and funders. Costs will be calculated from actual billable costs to the funder. Costs and benefits will be discounted at 4%. Cost-effectiveness and cost-utility will be calculated using the incremental costs and incremental benefits, with results presented as incremental cost-effectiveness and incremental cost-utility ratios. Bootstrapping will be undertaken to assess uncertainty, and discounting will be analysed through a one-way sensitivity analysis.

**Discussion:**

This pilot will make a novel contribution by trailing the use of a Synthetic Cohort Method to reduce the lengthy time horizons required in prosthetic HEE. The HEE will use a two-pronged approach whereby cost-utility and cost-effectiveness are simultaneously evaluated using the PROMIS instruments to inform a wide range of policy and investment decisions. Additionally, this pilot will be the first HEE of suction suspension systems for people with transtibial amputation and will therefore make an important contribution to the prosthetic evidence base.

## Background

Health Economic Evaluations (HEEs) seek to evaluate the costs and benefits of one healthcare intervention over another [1]. HEEs provide the necessary evidence of the cost–benefit of competing interventions to inform policy and investment decisions. In turn, those policy and investment decisions influence the type of healthcare available to consumers, such as the type of prosthetic interventions available to people living with limb-loss.

As prosthetic technology advances, a wide variety of high-cost and high-volume prosthetic interventions have become available to people living with limb-loss. For example, many people living with trans-tibial amputation use prostheses incorporating suction suspension where an airtight socket environment is created through the expulsion of air between the residual limb and the socket. There are two main types of suction suspension:

1. Passive suction (PS)—also known as suction suspension system—uses a sleeve and one-way value that allows air to be expelled from inside the socket environment and prevents air from entering.
2. vacuum-assisted suction (VAS)—also known as elevated-vacuum, sleeveless vacuum, and sub-atmospheric—uses a vacuum element, typically a mechanical pump, that actively draws air from the inside the socket environment [2–4].

VAS suspension provides more positive suspension compared to PS suspension [2, 3, 5] but, it comes at an increased cost compared to PS given the additional hardware (e.g., a mechanical pump) and maintenance required [6].

Whilst there have been some investigations into the benefits of VAS vs PS suspension systems for people living with trans-tibial amputation [2, 6], there have not been any HEEs [3, 7, 8]. In the absence of HEEs comparing the costs and benefits of these different prosthetic technologies, it is difficult for prosthetic funders to make decisions about which prosthetic technology they should fund at an individual user-level. Similarly, it is difficult for funding agencies to develop evidence-informed policies that guide funding decisions based on which of these prosthetic interventions provides the greatest cost–benefit.

While there is an important gap in the available HEE evidence, there are significant challenges to addressing this gap. A recent systematic review highlighted several method design issues affecting the quality and rigour of prosthetic HEEs [7]. For example, the benefits measured in many prosthetic HEEs (e.g., temporospatial parameters) are not sensitive to differences between competing prosthetic interventions [7], nor reflect what was important to prosthesis users and funders [9]. In addition, the time horizons over which the HEEs were conducted were too short to capture the costs or benefits of the intervention over its useful life [7]. For example, 1 year time horizons have been used in HEEs of prosthetic knees [10], even though a useful life of 5 years is well documented for the Micro-Processor Controlled prosthetic knees included in these studies [11, 12].

To address concerns with the benefit measures used to date, researchers have determined the benefits that are most important to prosthetic users and funders, and reported those in a 14-item Prosthetic Interventions Core Outcome Set (PI-COS) [9]. Further work has determined that the best way to measure the 14-items in the PI-COS is to use the Patient Reported Outcomes Measurement Information System (PROMIS) Physical Function with Mobility Aid short form and the PROMIS-29 + 2 [13]. This combination of PROMIS instruments [14–16] allows a HEE to take a two-pronged approach whereby clinical effectiveness and health utility are measured simultaneously, using an instrument that is likely sensitive to differences in prosthetic interventions [17].

Similarly, to address the time horizon challenge, we propose an innovative Synthetic Cohort Method where participants are recruited simultaneously into sub-groups reflecting the different years of the intervention post-fitting—a technique often used in epidemiological modelling of life-time risks [18–21]. A synthetic cohort is defined as a pooled data set constructed by combining multiple individual sub-groups spanning different periods of time of the life course [19], rather than data reflecting the experience of one cohort of participants over the life course. Assuming a prosthetic intervention with a useful life of three years [22, 23], participants in years 1, 2, or 3 of their prosthetic intervention could be recruited concurrently into sub-groups reflecting each year of a 3 year time horizon. Assuming these sub-groups were similar in terms of the parameters that significantly affect the costs and benefits (e.g., age, sex, or cause of amputation), participant data from each of the three sub-groups might be considered sufficiently representative to estimate the costs and benefits for an intervention over its useful life. This approach would allow the costs and benefits of the intervention to be estimated without the need to prospectively capture data across the useful life of the intervention and thereby shorten a 3-year data collection period down to 1-year. Considering that the useful life of some prosthetic interventions is up to 8 years (e.g., microprocessor controlled prosthetic knees), this innovative method has potential to transform the way we approach HEE.

While these two approaches may help solve the most important limitations of previous prosthetic HEEs, they are untested. As a result, there are several unknowns that justify a pilot study. For example, we do not know whether the PROMIS instruments are sensitive to differences in PS and VAS suspension for people using trans-tibial prostheses. Given the PROMIS instruments yield a score for each item bank, there are challenges interpreting the multiple ICERs. Similarly, we do not know how many participants would be required in each intervention arm given the likely difference in the outcome between interventions, and the variability of these data (effect size). Further, we do not know which demographic or clinical factors significantly influence the costs and benefits of these trans-tibial prosthetic suspensions systems and as such, it is not clear which of these factors should be matched between sub-groups during recruitment or controlled for statistically.

## Methods/Design

This protocol describes a pilot study that aims to trial an innovative method designed to compare the cost–benefit of PS and VAS suspension systems for people who use trans-tibial prostheses. What we learn from this pilot study will provide useful information about: implementation of the Synthetic Cohort Method, the measurement of benefits that are most important to prosthesis users and funders using two PROMIS instruments, and the usefulness of the aforementioned PROMIS instruments to report cost-effectiveness. That information can help guide the design of a larger-scale HEE into the future.

Given the aim of this study, the method has been sub-sectioned to report an observational study describing the costs and benefits of PS and VAS suspension in people living with trans-tibial amputation, and a subsequent HEE. Within that structure, we report key aspects of the HEE as recommended by the Consolidated Health Economic Evaluation Reporting Standards (CHEERS) checklist [24]; acknowledging there is variation in the sub-sectioning to remove unnecessary duplication between reporting of the observational study and the HEE.

### Observational study

#### Study setting

In Australia, prosthetic interventions are typically funded through one of three types of schemes with no out-of-pocket costs (e.g., financial contribution made by the prosthetic user):

- State Government funded (e.g., State Artificial Limb Schemes),
- Federal Government funded (e.g., the National Disability Insurance Scheme, NDIS),
- Third-party insurance schemes (e.g., Transport Accident Commission, TAC).

While every person has access to prosthetic care through one of these types of schemes, there are variations in the services funded between these schemes. For example, state Government schemes do not typically fund VAS suspension systems given the funding and policy constraints of those schemes. In contrast, federal Government and insurance schemes (e.g., NDIS and TAC) approve funding for a wider range of prosthetic services considered necessary for the prosthesis user to achieve their goals.

In the Australian setting, VAS suspension systems are almost exclusively funded by federal Government and insurance schemes and delivered by private prosthetic providers in the community. Hence, we believe this is a viable avenue for recruitment. While we acknowledge that prostheses with PS and VAS suspension may be provided by hospital-based services funded by a state Government scheme, we do not believe these avenues of recruitment will provide viable numbers of participants, nor provide comparable costings to the federal Government and insurance schemes given the different funding and policy constraints.

#### Participants

Participants in this investigation will be people:

- Living with unilateral trans-tibial amputation; irrespective of the cause of amputation or presence of comorbid health conditions,
- Living in the community > 12-months post-amputation,
- Whose prosthetic care is funded through a federal Government or insurance agency scheme given state funding schemes do not typically fund VAS suspension,
- Who receive their prosthetic care from from a private prosthetic service provider in the community,
- Who use a trans-tibial prosthesis that:
  - Includes either PS or VAS suspension,
  - Was fitted within the preceding three years irrespective whether it is the first or a subsequent prosthesis for the participant; where fitting is defined as the point of prosthesis provision that led to the acquittal of the service and invoicing to the prosthetic funder,
- Aged ≥ 18 years, and
- Able to complete questionnaires in English.

All participants will provide written consent as a condition of the Human Research Ethics Committee of La Trobe University.

#### Patient and public partnership

This protocol is part of a wider program of research to explore innovative solutions to HEE method design challenges in prosthetics. Prosthesis users and funders were contributors to a foundational publication which explored the benefits that are most important to prosthetic users and funders and were purposefully engaged through a Delphi consensus process and Expert Panels [9]. The results of this study influenced the choice of benefit measure and instruments described in this protocol. In designing this protocol, we have engaged with prosthetic funders to understand their most pressing policy and investment challenges and with prosthetic experts to gain knowledge of the technology and typical costs and proposed benefits of each intervention arm.

#### Interventions

For the purpose of this study, PS and VAS suspension will be defined in accord with a published clinical guideline [2]:

- PS suspension: the air-tight environment is created through the passive expulsion of air. For example, using a one-way value. There is no mechanical mechanism to actively extract the air.
- VAS suspension: the air-tight environment is created through active extraction of air, using a mechanical device (e.g., mechanical pump). This type of suspension requires an additional component, being a mechanical or electronic pump, that is attached to the prosthesis, either via the socket or the pylon and/or foot mechanism.

All interventions will be prescribed and fitted by a qualified prosthetist holding current certification through the Australian Orthotic Prosthetic Association. Concurrent treatments (e.g., use of walking aids, physiotherapy services) will be permitted given this study will take a pragmatic approach, seeking to measure the effectiveness of PS and VAS suspension in real-life. To help understand the impact of such co-interventions, detailed demographic and clinical data will be recorded.

#### Demographic and clinical data, benefit and cost measurement

A range of demographic (e.g., age, sex) and clinical (e.g., cause of amputation, presence of comorbid health conditions, use of co-interventions such as walking aids) data will be collected using a self-developed REDCap survey (Vanderbilt University, Tennessee) [25]. These survey questions will be based on demographic and clinical characteristics reported in large population-based studies and registries [26, 27] and will be used to characterise prosthesis users and explain variation in the costs and benefits observed. Additionally, a range prosthetic service provider data will be collected (e.g., number of employees, geographic location) using a self-developed REDCap survey (Vanderbilt University, Tennessee) that will allow characterisation of the prosthetic service providers and support the generalisability of the results.

Benefits will be measured using a REDCap survey (Vanderbilt University, Tennessee) including the PROMIS Physical Function with Mobility Aid short form and the PROMIS-29 + 2 [14–16]. These instruments have been selected given they provide the greatest coverage of the PI-COS [13]—which describes the benefits that lower limb prosthetic users and funders consider the most important [9]—and allows a two-pronged HEE that will simultaneously measure effectiveness and estimate utility through a preference-based utility scores (e.g., quality adjusted life years, QALYs) [15]. The PROMIS survey questions will be downloaded from the REDCap Shared Library to avoid the risk of errors [25, 28].

All costs billed to the federal Government or insurance scheme will be reported, including, as illustrative examples:

- Clinical costs associated with provision of the prosthesis (e.g., hourly rate charged for casting, modification, fitting),
- Technical costs associated with provision of the prosthesis (e.g., hourly rate charged for building the prosthesis),
- Component-related costs (e.g., the vacuum-pump, prosthetic foot, pylon or prosthetic socks),
- Clinical and technical costs related to ongoing support and services (e.g., review appointment, education and training session, fit adjustments),
- Non-clinical costs associated with managing patient care (e.g., hourly rate charged for report writing, patient education, case conferencing, liaising with other health professionals),
- Non-clinical costs associated with provision of services (e.g., administrative charges for scheduling appointments, ordering componentry).

The funder perspective adopted in this HEE—which is detailed in the latter part of the method—informs the identification and measurement of costs. Given this perspective, the list of costs is not exhaustive, but consists of all costs that will be billed to the funder for the intervention. These billable costs will be reported for each participant based on their allocated sub-group that reflects a year of the intervention since fitting. Billable costs will be extracted from the invoices provided by prosthetic service providers. Researchers will enter these data into a custom spreadsheet. Micro-costing will be used, with costs categorised according to their type (e.g., initial prosthesis, clinical service, componentry) to allow the exploration of cost variance [1]. The sum total will be calculated for each sub-group (i.e., Year 0–1), each intervention arm (e.g., PS suspension) and for each cost category. Categorising costs in this way will help explore the factors that influence the incremental cost variance, ICER and ICUR between the two intervention arms. For example, whether VAS componentry cost vs clinical service costs are the largest contributor to cost variance when compared to PS costs.

Detailed information about the prosthetic intervention (e.g., socket design, type and model of prosthetic foot) will also be reported. Prosthetic service providers will complete a standardised inventory of the participant’s prosthesis design and componentry.

#### Synthetic cohort method

The Synthetic Cohort Method will include PS and VAS suspension interventions, each including sub-groups whereby the costs and benefits will be estimated over 3-years, using just 1-year of data collection. Within each intervention arm there will be three sub-groups representing trans-tibial prosthesis users in each year of the intervention since fitting; that is: Year 0–1 (0 to ≤ 12 months), Year 1–2 (> 12 to ≤ 24 months) and Year 2–3 (> 24 to ≤ 36 months).

Participants will be assigned to one of the sub-groups based on the type of suspension used (i.e., PS or VAS) and the year(s) of the intervention since fitting (e.g., Year 2–3). For example, a participant who has been using PS suspension system for 2 years and 6 months will be allocated to PS, Year 2–3 (PS_Y2-3_) group (Table 1, dark grey shaded cell).

**Table 1:**
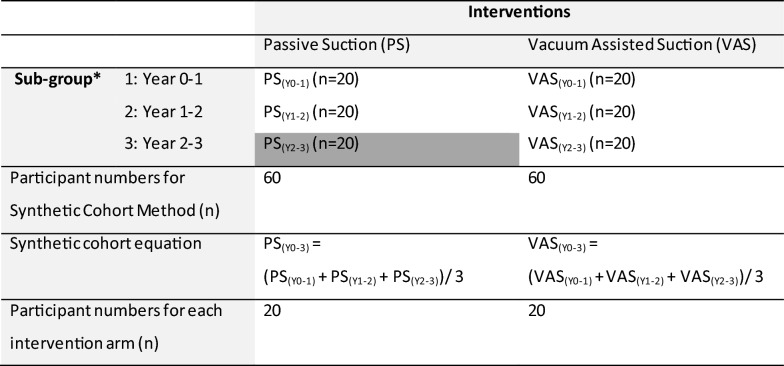
Participants in the Synthetic Cohort Method, for each intervention arm and sub-group

^*^Based on year since prosthetic fitting

The number of participants in each of the PS and VAS suspension intervention arms was calculated based on a minimum important change (MIC) T-score of 5 points on the PROMIS instruments, two-sided significance level of 5% (α = 0.05) and power of 80% [29], including a 25% accommodation for loss-to-follow-up. While there is no evidence for a MIC for the PROMIS instruments specific for trans-tibial prosthesis users, recent evidence indicates the MIC is likely between 4 and 10.5 T-Score points in similar populations (i.e., people living with stroke or foot amputation) [30, 31]. Given the variation in the MIC between studies, a 5 T-score was adopted based on the results of a recent systematic review [32] and recommendations that a half SD is used (equivalent to 5 T-score points in the PROMIS) where there is no empirical evidence [33]. To achieve 20 participants for each of the PV and VAS suspension intervention arms, a total of 60 participants—20 in each sub-group based on year of the intervention since fitting—are required (Table 1).

#### Procedure

To facilitate the recruitment of participants, we will collaborate with the largest prosthetic service providers in each Australian state or territory based on the number of practitioners employed (a proxy for number of clients living with trans-tibial amputation). Using the Australian Orthotic Prosthetic Association online Find a Practitioner tool [34], researchers will rank prosthetic service providers in each state or territory based on the number of practitioners employed. Researchers will then write to the largest facility in each state or territory introducing the study and invite them to a one-on-one meeting to explain the study, address questions, and introduce a standardised statement that covers the terms of their involvement. Prosthetic service providers will have 4 weeks in which to return a signed copy of the standardised statement. During that time, they’ll have opportunity to follow up with any further questions about the study. A reminder email will be sent to the prosthetic service provider 1 week, and 1 day before the end of the 4-week period.

Collaborating prosthetic service providers will screen their database to identify trans-tibial prosthesis users that meet the aforementioned inclusion criteria. Assuming only small numbers of PS and VAS suspension users will be identified, each prosthetic service provider will email all prospective participants inviting them to contact the research team to express interest in participating. Email reminders will subsequently be sent 2 weeks later.

Prospective participants can then discuss participation through a scheduled phone call with the research team. In this phone call, the researcher will confirm their eligibility against the inclusion criteria and obtain an email address and phone number. The researcher will then email a Participant Information Statement and consent form. Prospective participants will be followed up by email 1 and 2 weeks after distribution of the consent form. Where there is an error in the email address, researchers will contact participants using their provided phone number.

Those people interested in participating will return a signed consent form; after which, participants will be scheduled for the collection of three types of data: demographic and clinical data, benefit data and cost data. Data will be collected for each participant at two time points: 1) demographic and clinical data at enrolment, and 2) benefit and cost data collected at the end of the participant’s sub-group based on year of the intervention since fitting (e.g., end of Year 0–1) and review of demographic and clinical data.

This two-staged approach to collecting data will help minimise the size of the final survey and build early engagement in the study with a view to minimising study drop out. At the point of enrolment into the study, participants will be at different time points since their final prosthetic fitting. To manage the collection of data at different time points for each participant, the date for the end of each participant’s sub-group based on year of the intervention since fitting will be used to schedule administration of the final survey.

At the first data collection time-point (i.e., enrolment), participants will be sent an email with a link to the online REDCap survey [25] that consists of demographic and clinical questions. A follow up email will be sent 1- and 2-weeks later to those yet to complete the survey.

At the second data collection time-point (i.e., end of participant’s sub-group year), participants will be emailed with a link to a REDCap survey 4 weeks prior. An email reminder will occur at 1- and 2-weeks post distribution where participants have not yet responded. That survey will include the PROMIS Physical Function with Mobility Aid short form and the PROMIS 29 + 2 survey instrument. In addition to completing the PROMIS survey instruments, participants will review their demographic and clinical data, and note any changes since enrolment in the study, to allow confirmation of eligibility (e.g., participant hasn’t undergone a more proximal amputation) and determine whether their health status has changed since the first data collection time-point.

Following the participant’s completion of the final survey, cost data will be collected for each participant directly from their prosthetic service provider. The research team will email prosthetic service providers with a request for copies of all invoices for the relevant participant.

#### Data synthesis

Descriptive statistics appropriate to the data type will be used to characterise demographic and clinical factors of the PS and VAS interventions, as well as sub-groups for each intervention arm.

In accord with PROMIS recommendations [35], the PROMIS Physical Function with Mobility Aid short form and PROMIS 29 + 2 will be scored using the HealthMeasures Scoring Service [36]. T-Scores will be transformed from theta values for each of the 8 item banks of the PROMIS-29 + 2 and the PROMIS Physical Function with Mobility Aid short form. These 9 results will be reported for each sub-group and for both the PS and VAS intervention arms. Average costs (mean ± standard deviation) will be reported for each sub-group within both the PS and VAS suspension intervention arms. Table 2 provides the equations for calculating the mean costs and mean benefits for each intervention arm using the Synthetic Cohort Method.

**Table 2:** Data analysis for the costs and the benefits (e.g., PROMIS Physical Function with Mobility Aid short form and PROMIS 29 + 2 results) for each intervention arm, each consisting of three sub-groups

		Interventions	
		Passive-suction (PS)	Vacuum-assisted suction (VAS)
Sub-groups*	Year 0–1 (µ) Costs and Benefits	PS_(Y0-1)_/n	VAS_(Y0-1)_/n
	Year 1–2 (µ) Costs and Benefits	PS_(Y1-2)_/n	VAS_(Y1-2)_/n
	Year 2–3 (µ) Costs and Benefits	PS_(Y2-3)_/n	VAS_(Y2-3)_/n
µ Costs (Y0-3)		[PS_(Y0-1)_/n] + [PS_(Y1-2)_/n] + [PS_(Y2-3)_/n]/3	[VAS_(Y0-1)_/n] + [VAS_(Y1-2)_/n] + [VAS_(Y2-3)_/n] / 3
µ Benefits (Y0-3)		[PS_(Y0-1)_/n] + [PS_(Y1-2)_/n] + [PS_(Y2-3)_/n]/3	[VAS_(Y0-1)_/n] + [VAS_(Y1-2)_/n] + [VAS_(Y2-3)_/n] / 3
ICERs/ICUR		VAS_(Y0-3)_ µ Cost—PS_(Y0-3)_ µ Cost VAS_(Y0-3)_ µ Benefits—PS_(Y0-3)_ µ Benefits	

### Health economic evaluation

The HEE will utilise data collected from the observational study previously described. This method was chosen in contrast to meta-synthesis and modelling approaches given the lack of data in the wider literature, especially over the useful life of the interventions. Given the method for the observational study includes much of the detail required of any HEE (e.g., study setting, interventions and collection method for benefit and cost data) a simplified account of the HEE method follows, covering the additional information required by the CHEERS [37].

#### Perspective

A healthcare payor (i.e., prosthetic funder) perspective will be used given that federal Government and insurance agencies in Australia need evidence about which of these competing interventions provide the greatest cost–benefit to inform both individual funding decisions as well as guide the development of evidence-informed policies.

#### Time horizon

The time horizon for this HEE is 3-years given this is the accepted useful life of PS and VAS suspension by Australian prosthetic funders, as reflected in the policies that restrict earlier replacement unless there is a medical need [22, 23].

#### Calculating incremental costs and incremental benefits

The mean costs for each intervention arm will be drawn from the observational study and will therefore reflect actual costs billed to the federal Government or insurance scheme. Costs will be reported in Australian dollars in the year for which they are accrued and a discount rate of 4% will be applied in accord with the Australian Government Department of Treasury and Finance technical guidelines on economic evaluation recommendation [38]. Incremental cost will be calculated as the mean cost of VAS suspension minus the mean cost of PS suspension.

Similarly, the benefit data for the HEE will be drawn from the observational study and will consist of mean benefits for each intervention arm as measured by the PROMIS instruments. For effectiveness, mean T-scores calculated from the Physical Function with Mobility Aid short form and the PROMIS 29 will be used given it provides the most extensive coverage of the PI-COS that describes the outcomes that are important to prosthetic users and funders [9, 13]. This will provide a singular effectiveness result (e.g., T-scores) for the Physical Function with Mobility Aid short form and 8 effectiveness results calculated from the PROMIS-29 representing the 7 item banks and 1 pain scale of this instrument. For utility, the PROMIS utility score value set (i.e., the PROPr) will be calculated from the PROMIS-29 + 2 which includes 7 item banks and 2 cognitive function questions [39]. This utility score value set has been calculated based on the stated preferences of the US population [15, 16], noting Australian population preference data have not been recorded. To calculate the utility score, the T-scores calculated from the PROMIS 29 + 2 will be converted to the PROPr preference-based score that represents utility based on the available R code [35]. Incremental benefits (e.g., effectiveness and utility) will be calculated as the mean benefits of VAS suspension minus the mean benefits of PS suspension.

The formula for calculating sub-group means, intervention arm means, and incremental costs and benefits are provided in Table 2. Results will be presented as incremental costs (e.g., AUD$), incremental effectiveness (e.g., T-score units) and incremental utility (e.g., QALYs), including means for each sub-group of the intervention arms.

#### Cost-effectiveness and cost-utility analysis

Incremental cost effectiveness ratios (ICERs) and an incremental cost utility ratio (ICUR) will be calculated. The 9 ICERs will be calculated—from the 2 PROMIS instruments—as the difference in mean cost (incremental cost), divided by the difference in mean effectiveness (incremental effectiveness, measured as incremental change in T-scores) between the PS and VAS suspension intervention arms. A singular ICUR will be calculated using the difference in mean utility (incremental benefits) [15] (Table 2).

#### Sensitivity analysis

Decision uncertainty will be explored through a probabilistic sensitivity analysis that will assess the degree to which variation in cost and benefit estimates affect the relative ICER and ICUR [40]. Bootstrapping will be used through 1000 replications of the original data to assess the effect of variations in the estimates, with selection of the bootstrapped estimates based on good practice guidance regarding the likely distribution of parameters [40]. The probabilistic sensitivity analysis results for the incremental costs and incremental benefits will be plotted on an incremental cost-effectiveness plane to demonstrate cost-effectiveness, dominance and dominated outcomes for one intervention in comparison to the other [40, 41]. From this scatter plot, a cost-effectiveness acceptability curve (CEAC) will be constructed to visually represent the probability that one of the interventions is more cost-effective than the alternative, across a range of willingness to pay thresholds [41]. Noting the use of a 4% discount rate [38] for costs and benefits, the effect of varying this rate (e.g., 0%, 3%, 7% and 10%) on the relative ICER and ICUR will also be analysed using simple one-way sensitivity analysis.

### Ethics and dissemination

The study will be undertaken in accord with the National Statement Ethical Conduct of Human Research [42] following approval by the La Trobe University Human Research Ethics committee. Data will be securely stored in accord with current standards for security and data privacy, and all personal data of prospective and enrolled participants will be password protected and only accessed by authorised persons.

Publications planned from this study include a peer-reviewed journal article. In addition, the work will be disseminated through a research translation article and conference presentations that explore the feasibility of the Synthetic Cohort Method for addressing long time horizons and the potential use of the PROMIS instruments for future prosthetic HEEs.

## Discussion

This protocol describes a pilot study to compare the cost–benefit of PS and VAS suspension systems for people who use trans-tibial prostheses. Importantly, it will also allow for the trial of several innovations to the method design that will guide a larger-scale HEE into the future. Specifically, this pilot study will trial:

- Use of the Synthetic Cohort Method to address the lengthy time horizons required for HEEs of prosthetic interventions. To our knowledge this method has not been used in HEEs using trial or observational data, and the potential benefit of this method warrants investigation through a pilot study.
- Measuring benefits using the PROMIS Physical Function with Mobility Aid short form in conjunction with the PROMIS 29 + 2, that capture the outcomes most important to prosthesis users and funders.
- Trial the reporting of 9 ICERs calculated from the two PROMIS instruments and how these detailed cost-effectiveness results may inform policy and investment decisions. For example, specific ICER results may be used to inform individual funding decisions related to clinical goals (e.g., use of the pain item bank ICER where a prosthesis user has a goal to reduce the experience of pain).

In addition to trialing the innovative method, the results of this study will make an important contribution to the body of prosthetic HEE knowledge, specifically in the area of trans-tibial prosthetic suspension systems in which no prior HEEs exist [3, 7, 8].

Whilst the trial will focus on assessing method implementation (e.g., participant recruitment across 3 sub-groups of an intervention arm), this pilot study will also collect detailed demographic and clinical data that will help identify which factors influence costs and benefits for people using PS and VAS suspension systems. Should this pilot establish the feasibility of implementing a Synthetic Cohort Method, then future exploration is recommended to investigate of the extent to which comparable sub-groups can be established given the factors that influence costs and benefits.

## Data Availability

No datasets were generated or analysed during the current study.

## Abbreviations

CEAC: Cost-effectiveness Acceptability Curve
CHEERS: Consolidated Health Economic Evaluation Reporting Standards
HEE: Health Economic Evaluation
ICER: Incremental Cost-effectiveness Ratio
ICUR: Incremental Cost-utility Ratio
MIC: Minimum Important Change
NDIS: National Disability Insurance Scheme
PI-COS: Prosthetic Interventions Core Outcome Set
PROMIS: Patient-Reported Outcomes Measurement Information System
PS: Passive suction
TAC: Transport Accident Commission
VAS: Vacuum assisted suction
